# Overexpression Levels of *LbDREB6* Differentially Affect Growth, Drought, and Disease Tolerance in Poplar

**DOI:** 10.3389/fpls.2020.528550

**Published:** 2020-11-11

**Authors:** Jingli Yang, Hanzeng Wang, Shicheng Zhao, Xiao Liu, Xin Zhang, Weilin Wu, Chenghao Li

**Affiliations:** ^1^State Key Laboratory of Forest Genetics and Breeding, Northeast Forestry University, Harbin, China; ^2^School of Pharmacy, Harbin University of Commerce, Harbin, China; ^3^Agriculture College of Yanbian University, Yanji, China

**Keywords:** poplar, drought stress, dehydration responsive element binding transcription factor, dwarf, disease tolerance

## Abstract

The application of drought stress-regulating transcription factors (TFs) offers a credible way to improve drought tolerance in plants. However, many drought resistant TFs always showed unintended adverse effects on plant growth or other traits. Few studies have been conducted in trees to evaluate and overcome the pleiotropic effects of drought tolerance TFs. Here, we report the dose-dependent effect of the *Limonium bicolor LbDREB6* gene on its overexpression in *Populus ussurensis*. High- and moderate-level overexpression of *LbDREB6* significantly increased drought tolerance in a dose-dependent manner. However, the OE18 plants showed stunted growth under normal conditions, but they were also more sensitive to *Marssonina brunnea* infection than wild type (WT) and OE14 plants. While, OE14 showed normal growth, the pathogen tolerance of them was not significantly different from WT. Many stress-responsive genes were up-regulated in OE18 and OE14 compared to WT, especially for OE18 plants. Meanwhile, more pathogen tolerance related genes were down-regulated in OE18 compared to OE14 and WT plants. We achieved improved drought tolerance by adjusting the increased levels of exogenous *DREB* genes to avoid the occurrence of growth reduction and reduced disease tolerance.

## Introduction

Drought is among the most serious environmental stressors resulting in substantial damage annually to the global agricultural and forestry industries (Kudo et al., 2017). Thus, improving drought tolerance of plants is urgently needed to stabilize the global productivity of crops. Vascular plants have evolved complex molecular strategies to cope with water deficit (Lata and Prasad, 2011; Li et al., 2014). In particular, vascular plants mediate some stress responses to drought via transcription factors (TFs), which serve as master regulators of many stress-response genes (Singh et al., 2002; Nakashima et al., 2014). Therefore, TFs may comprise critical targets for transgenic engineering to modify the tolerances of plants to abiotic stressors (Quan et al., 2010). However, the application of TFs to improve drought tolerance frequently comes at the cost of introducing undesired phenotypes, such as dwarfism, decreased pathogen tolerance, and decreased grain yield (Lata and Prasad, 2011; Bhargava and Sawant, 2013; Cabello et al., 2014; Shavrukov et al., 2016). These negative effects constrain the practical utilization of drought-resistant transgenic plants based on TFs. Thus, to evaluate the drought tolerance conferred by TFs, it is necessary to simultaneously predict and avoid unintended effects in transgenic plants.

These TFs, especially the dehydration responsive element binding (DREB) factor family member, occur in many plant species and confer tolerance to abiotic stress (Lin et al., 2008; Liao et al., 2017). In *Arabidopsis thaliana* (L.) Heynh., the DREB subfamily is divided into six subgroups (A-1 to A-6) based on the structural characteristics of the proteins (Agarwal et al., 2017). Among the subgroups, A-1 and A-2 contain *DREB1* and *DREB2* TFs, which are known to be involved in stress responses to low temperature, drought, and high salt (Du et al., 2018). In *Arabidopsis*, *DREB2* positively regulates the expression of drought-response genes (Sakuma et al., 2006). Overexpression of Glycine max *DREB1* (*GmDREB1*) improved drought tolerance and responses to other abiotic stressors in transgenic *Arabidopsis* and wheat (Kidokoro et al., 2015; Zhou et al., 2020). Moreover, ectopic expression of *GhDREB1* from cotton yielded stronger tolerance to chilling in transgenic *Nicotiana tabacum* L. (tobacco) compared to the wild type (WT) (Shan et al., 2007).

DREBs of the A-6 subgroup have also been identified from several plant species, and their functions in stress responses and development have been characterized. For example, expression of *GhDBP2* is greatly up-regulated during drought, salt exposure, low temperature, and abscisic acid (ABA) treatments in the cotyledons of cotton plants (Huang et al., 2008), and overexpression of *CmDREB6* in chrysanthemum enhanced heat tolerance (Du et al., 2018). Similarly, *JcDREB*, an A-6 subgroup member from *Jatropha curcas* L., a biodiesel plant, was up-regulated by cold, salt, and drought stress, and overexpression of *JcDREB* in transgenic *Arabidopsis* enhanced salt and freezing tolerance (Tang et al., 2011). In tobacco, overexpression of *SsDREB*, an A-6 DREB from halophilic *Suaeda salsa* (L.) Pall., increased salt and drought tolerance compared to WT plants (Zhang et al., 2015).

Although DREBs of several subgroups are known to increase drought tolerance, they have also been shown to inhibit growth in transgenic plants (Huang et al., 2009). For example, *ZmDREB4.1* from maize, repressed cell division and constrained leaf extension and hypocotyl, petiole and stem elongation both natively in transgenic tobacco (Li et al., 2018). Likewise, overexpression of *AtDREB1A* in soybeans led to dwarfism and delayed flowering (Suo et al., 2016).

DREBs are also reported to be involved in the biotic stress signaling pathway. For example, the overexpression of *At DREB1* in *Solanum tuberosum* L. (potato) enhanced tolerance to the fungal pest, *Fusarium solani* (Mart.) Sacc. (1881) (Charfeddine et al., 2015). In contrast, in transgenic *Arabidopsis*, overexpression of *MsDREB2C* improved drought stress tolerance but caused greater sensitivity to the pathogenic bacterium, *Pst* DC3000 (*Pseudomonas syringae* Van Hall, 1904 pv. tomato DC3000), and to the pathogenic fungus, *Alternaria mali* Roberts (1914), compared to WT plants (Zhao et al., 2013). Therefore, a more comprehensive evaluation of DREBs is needed to assess their performance in transgenic plants under combined effects from abiotic and biotic stressors.

Although there are significant advances in our understanding of drought tolerance in trees, especially through model systems such as poplars, there remains a limited number of field-based studies on water stress in transgenic trees, particularly under natural conditions. With respect to DREBs specifically, there have been few studies on the A-6 subgroup in woody plants representing either model or non-model systems. In a study on apples, overexpression of an A-6 subfamily member, *MsDREB6.2*, was found to confer drought tolerance (Liao et al., 2017), similarly to its homolog in *Arabidopsis*, *RAP2.4* (Lin et al., 2008). This result suggests that A-6 DREBs may be involved in stress tolerance in woody plants as they are in herbaceous species. Nevertheless, data are largely lacking, and almost nothing is known about the negative effects of DREBs on growth in transgenic trees or the interactions of DREBs with the biotic stress response. Overall, more tree transgenesis is urgently needed to link physiology, systems biology, and field performance to the benefit of silviculture industries.

In the present study, we investigated the effect of different expression levels of *LbDREB6*, a DREB of the A-6 clade from *Limonium bicolor* (Bunge) Kuntze, on plant growth, drought tolerance, and pathogen susceptibility in transgenic *Populus ussuriensis* Komarov under natural water-deficient conditions. We found that moderate overexpression of *LbDREB6* increased drought tolerance but did not affect plant growth and pathogen susceptibility, while high levels of overexpression of *LbDREB6* caused stunted growth and increased sensitivity to pathogen infections compared to the WT. Our study provides a basis for using genetic modification to improve drought tolerance in poplar while avoiding other adverse traits such as growth inhibition and reduction of disease tolerance, which may have negative implications for woody crop plants.

## Materials and Methods

### Plant Materials and Growth Conditions

*Populus ussuriensis* clone Donglin plants were grown *in vitro* on 1/2 MS semi-solid medium, with 0.6% w/v agar and 2% w/v sucrose, under irradiation of 16 h-light/8 h-dark cycles with PPFD of 46 μmol m^–2^⋅s^–1^ at 25°C. The cuttings were subcultured at 4-week intervals.

### Vector Construction and Populus Transformation

The coding region of *LbDREB6* (Ban et al., 2011) was fused into ProkII vector under the control of CaMV35S promoter. The binary vector ProkII carried the gene encoding neomycin phosphotransferase as a selection marker. The vector was introduced into *Agrobacterium* strain EHA105 prior to transformation by leaf discs using the method as described by Zhao et al., 2017. Transformants were selected on media containing 50 mg/L kanamycin. All transgenic plants were confirmed by genomic PCR and by mRNA qRT-PCR. qRT-PCR was used to quantify DREB6 transcripts using a conserved sequence (F: TAAGTGGGTGGCTGAGAT; R: CCTTCGGAACCGAATACTG). The *P. ussurensis PuActin* gene (GeneBank accession number: MH644084) was used as the reference gene. All the primers are listed in Supplementary Table 1.

All regenerated WT and transgenic plantlets with well-developed leaf and root systems were transferred to the same size pots containing an autoclaved sand and soil mixture (1:3 v/v) at the same growth period. Potted plants were covered with transparent plastic to maintain high humidity and incubated in a growth chamber at 21°C under a 16 h photoperiod for 2 weeks, and then the cover was removed. The growth characterization was recorded after 3 months. The high-level *LbDREB6* overexpression lines (OE18, OE32, and OE35) and moderate-level *LbDREB6* overexpression lines (OE10, OE14, and OE30) plants were used in the following assay.

## Western Blotting Analysis

Western blotting was performed as described by Chen et al. (2008). An anti-LbDREB6 polyclonal antibody (goat anti-mouse biotinylated immunoglobulin) was used to recognize LbDREB6. The antibody was prepared by the Institute of Genetics and Developmental Biology, Chinese Academy of Sciences. Immunoreactive polypeptides were visualized by using the Western ECL blotting substrate BeyoECL Moon (Beyotime, China). Imaging was performed using a LAS-4000 imaging system (Fijifilm Life Science, United States).

### Growth Characteristics

Hand cut sections of the youngest fully developed leaves of WT, OE18, and OE14 plants that were cultured in a flask were cut transversely at the middle of the leaf and fixed with 4% formaldehyde in phosphate-buffered saline with 0.5% TritonX-100 for 4 h at 23°C in a tube roller. After three 10-min washes, sections were mounted in 0.1 mg⋅mL^–1^ Calcofluor White in water and imaged using Olympus microscopy (SZX7). Perimeters of leaf parenchyma cells were manually traced in Olympus Fluoview software and plotted. Using the same software, the area of hand cut sections of WT and transgenic plant stems was calculated. The same method was used for cross sections of the stems. For scanning electron microscopy (SEM), stem segments of 1-year-old plants grown in a greenhouse (OE18 and OE14) were glued onto aluminum stubs and placed on a chamber stage that had been precooled to −120°C. The samples were viewed using a VP-SEM instrument (S-3500N, Hitachi, Tokyo, Japan). After the regenerated plantlets were transferred to a greenhouse for 2 months, their height and leaf width of were investigated. Meanwhile, the gibberellic acid (GA) content of shoot tip samples was analyzed following the protocol instructions of the Plant Gibberellic Acid, GA, ELISA kit (San Diego, CA, United States). For GA treatment, plantlets transplanted for 2 months in a greenhouse under natural sunlight were sprayed daily with 100 μM GA_3_ for 15 days. After 1 month, the height of WT, OE14, and OE18 plantlets was measured. For each experiment, 10 samples were used with three replicates. Data are presented as means of three biological replicates and error bars represent ±SD.

### Drought Tolerance Assay

For plant growth analysis under drought stress, 6-month-old plants grown in pots in the greenhouse were used to conduct experiments. WT, OE14, and OE18 plants were grown under drought conditions for 2 weeks, and re-watered under normal conditions for a 2-week recovery period. The relative water content (RWC,%), chlorophyll content and gas exchange were investigated under drought stress. The growth height of WT and transgenic plant lines were also investigated after re-watering for 2 weeks. Additionally, we cultured the sterile cutting seedling in a flask under 7% PEG6000 treatment for 2 days. All the experiments were repeated three times.

### Physiological and Biochemical Analysis

Before conducting the following experiments, plants were subjected to drought contains for 5 days in a greenhouse. Leaf RWC and chlorophyll were extracted and analyzed according to a previously published method (Wang et al., 2016). Leaf gas exchange was measured using a portable open gas exchange system (Li-6400; Li-Cor, Inc., Lincoln, NE, United States) equipped with a light source (Li-6200-02B LED; Li-Cor). The transpiration rate (Tr), stomatal conductance (Gs), and net CO_2_ assimilation were measured in mature leaves between 0 and 5 days after drought stress. Environmental conditions in the leaf chamber consisted of a photosynthetic photon flux density of 1,400 μmol m^–2^⋅s^–1^, an air temperature of 25°C, and an ambient CO_2_ concentration of 400 μmol mol^–1^. Leaf malondialdehyde (MDA) content was determined as described by Chen et al. (2008). The electrolyte leakage (EL,%) was used to estimate cellular membrane stability, which was detected following the methods described by Gu et al. (2017). Leaf hydrogen peroxide levels were determined as described by Yin et al. (2010). The absorption of the supernatant was read at 390 nm, and the content of H_2_O_2_ was quantified based on the standard curve. All the experiments were repeated three times.

### Inoculation of Poplar With Marssonina Brunnea

Individuals of approximately equal size from WT, OE10, OE14, OE30, OE18, OE32, and OE35 lines after transplantation for 2 months in a growth chamber (25°C temperature, 70% humidity, 16 h/8 h light/dark cycle) were used for inoculation experiments. Plants were inoculated by thoroughly spraying a spore suspension of *M. brunnea* (±10^5^ spores mL^–1^) onto the abaxial leaf surfaces according to the method used by Ullah et al. (2017). Immediately after spraying, each plant was covered with a polyethylene terephthalate bag to maintain high humidity; this was kept in the dark to facilitate spore germination. After 24 h, the bags were removed. Control seedlings were sprayed with distilled water. Leaf infection grade was set according to Zhao et al. (2017). All the experiments were repeated three times.

### Transcriptome Analysis

We performed transcriptome analysis on two groups. One group was the fully expanded leaves, respectively, collected from WT and transgenic plant lines (OE14 and OE18) after drought treatment for 7 days in a greenhouse, and the other group was the apical meristem with two unexpanded young leaves which were harvested from WT, OE14, and OE18 plants after being inoculated with the pathogen *M. brunnea*, for 2 days; samples were immediately frozen in liquid nitrogen for RNA extraction. For each sample, ∼20 μg total RNA of each sample was sent to Annoroad Gene Technology Co., Ltd., Beijing, China for high throughout Illumina HiSeq 4000 sequencing (Illumina, San Diego, CA, United States). Each sample was conducted with three biological replicates and three technical replicates. The clean reads were mapped to *P. trichocarpa* mRNA reference sequence using TopHat (ver. 2.0.9) software (Trapnell et al., 2009). Transcript expression level is measured as fragments per kilobase of transcript per million mapped reads by Cufflinks (Trapnell et al., 2010). The reads per kilobase million (RPKM) value was used as the threshold for judging whether the gene was expressed; | log_2_FC| >1.0, *P* < 0.01, and a false discovery rate (FDR) < 0.01 were used as a threshold for the differential expression of genes to screen for DEGs. The DEGs and their encoded proteins were annotated by comparing them against NCBI, the Swiss-Prot database, NCBI non-redundant nucleotide sequence database, and non-redundant protein database. Then, the DEGs were imported into the Blast2 GO program (Götz et al., 2008) to identify gene ontology (GO) terms. Singular enrichment analysis in agriGO database^1^ was performed to identify significantly enriched GO terms. KEGG Orthology-Based Annotation System 2.0 (KOBAS,^2^) was utilized to identify significantly enriched pathways (Wu et al., 2006; Xie et al., 2011).

### qRT-PCR Analysis

RNA was extracted from leaves of WT and transgenic lines of *Populus* exposed to drought stress for 7 days. Randomly selected 17 drought responsive genes in OE14 and OE18 compared to WT plants and six genes related to drought stress in OE18 compared to OE14 plants were verified by qRT-PCR, respectively. The primers used are listed in Supplementary Table 1. Furthermore, the selected 14 genes related to disease tolerance (*PP2C, LRR-1, RPS2, RPM1, PYL, ABP19a, WRKY49, WRKY9*, and *NR*) and *GA20ox1* were verified by qRT-PCR. All the experiments were repeated three times. The primer sets used in this study to validate transcriptome data are given in the Supplementary Table 2. The raw sequence data of drought stress and pathogen infection RNA-seq experiments have been deposited in the Gene Expression Omnibus with the accession numbers GSE139373 and GSE120118, respectively.

### Data Analysis

Statistical testing was performed with IBM SPSS Statistics 21 (IBM Corporation, Armonk, NY, United States). The data were tested by Student’s *t*-test (^∗^*P* < 0.05 or ^∗∗^*P* < 0.01).

## Results

### Effect of High-Level and Moderate-Level LbDREB6 Overexpression on Growth in Transgenic Populus

**FIGURE 1:**
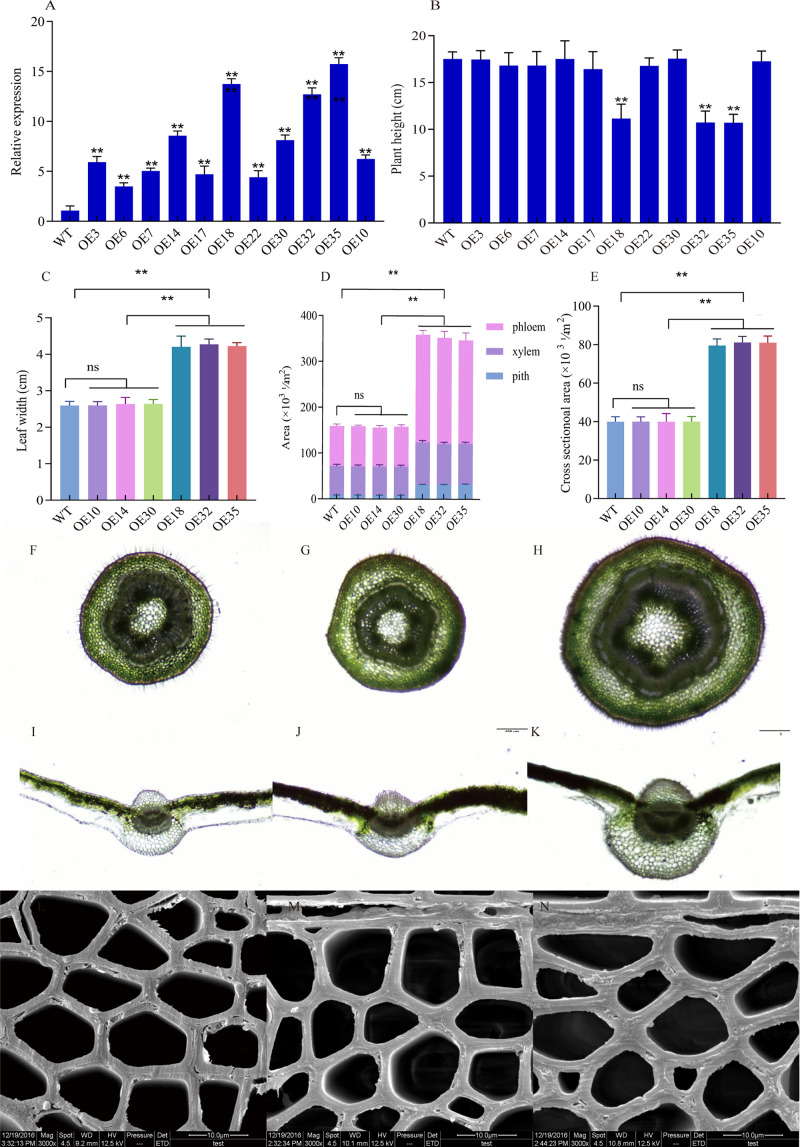
Growth characteristics, cross-section, and scanning electron microscopy (SEM) observation of high-level (OE18) and moderate-level (OE14) *LbDREB6* overexpression lines and wild type (WT) *P. ussuriensis* plants. **(A)** qRT-PCR validations of OE18, OE14, and WT plants. *PtrActin* was used as an internal control. Data are presented as means of four biological replicates, and error bars represent ±SD. **(B)** Heights of OE18, OE14, and WT plants grown in greenhouse for 2 months. **(C)** Leaf widths of WT, OE18, and OE14 plants. **(D)** The quantification of sectioned areas representing bark, wood, and pith regions. **(E)** Midrib cross-sectional areas of WT, OE18, and OE14 plants. Samples were collected at 1 cm from the stem foundation. Cross-section of the stem in WT **(F)**, OE14 **(G)**, and OE18 **(H)** plants. Cross-section of the leaf midrib-xylem in WT **(I)**, OE14 **(J)**, and OE18 **(K)** plants. SEM observation of cell walls in WT **(L)**, OE14 **(M)**, and OE18 **(N)** plants. For **(B–E)**, each value represents the mean of 20 plants with three biological replicates, and error bars represent ±SD. Asterisks indicate significant differences, *P < 0.05 and **P < 0.01.

A total of 11 independent *LbDREB6* overexpressing transgenic poplar lines were obtained by *Agrobacterium*-mediated genetic transformation and were analyzed through PCR. All lines had the expected 1,071 bp PCR product for the *LbDREB6* gene (Supplementary Figure 1A). The qRT-PCR results indicated that the expression levels of *LbDREB6* in transgenic plants were significantly higher than that in WT plants (Figure 1A). After transplantation for 2 months, the phenotyping experiment demonstrated that plants of most lines that had overexpression levels greater than 10 times that of WT plants were significantly shorter. In comparison, other transgenic lines with overexpression levels lower than 10 times that of the WT showed normal growth (Figure 1B). We chose three relatively moderate-level (OE10, OE14, and OE30) and three high-level (OE18, OE32, and OE35) *LbDREB6* overexpression lines to conduct the following assays (Supplementary Figure 1B). The western blotting analysis using an anti-LbDREB6 antibody revealed that OE18 had a high LbDREB6 protein expression compared to OE14, which was consistent with the RT-PCR result (Supplementary Figure 1C).

Leaf width of OE18 plants was significantly larger than that of WT plants (Figure 1C), while the leaf width was not considerably different between OE14 and WT plants (Figure 1C). Leaf and stem sizes are dependent on both the number and the size of cells in the organ (Zhou et al., 2013). Cross sections of stems revealed that the primary and secondary xylem rings of OE14 (Figures 1D,G) were not significantly different from WT plants (Figures 1D,F), while OE18 stems (Figures 1D,H) increased significantly compared to the WT plants (Figures 1D,F). As quantified by the cross-sectional area measurements, the leaf veins of OE14 (Figures 1E,J) was not significantly different from WT plants (Figures 1E,I), but the leaf veins of OE18 (Figures 1E,K) were 40∼50% larger than the WT plants (Figures 1E,I). SEM observation showed that cell walls of OE14 (Figure 1M) were not significantly different from WT plants (Figure 1L). However, the cell walls of OE18 (Figure 1N) were considerably thicker than WT plants (Figure 1L).

### Comparison of High-Level and Moderate-Level LbDREB6 Overexpression on Drought Tolerance

**FIGURE 2:**
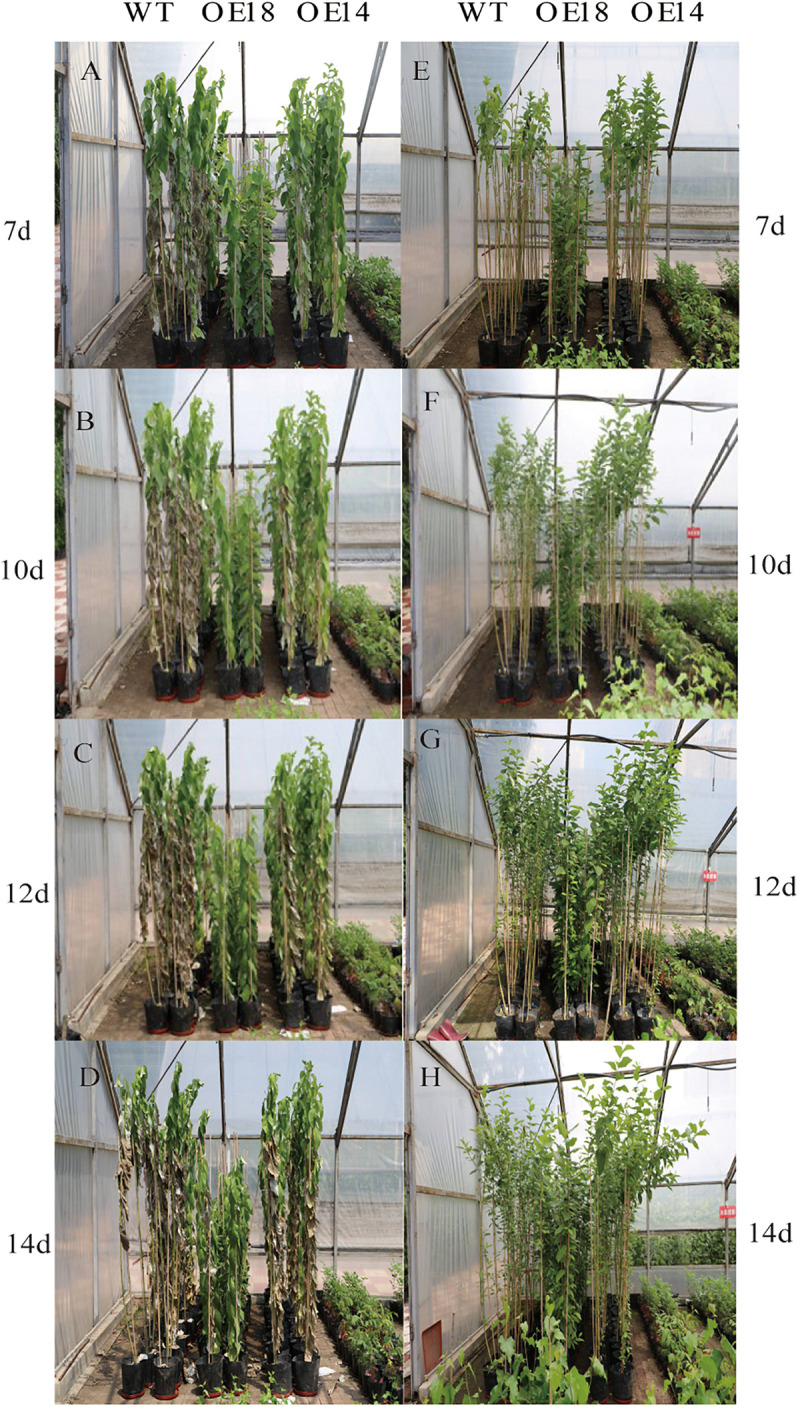
Phenotypic observation of WT and transgenic plant lines under drought stress for **(A)** 7d, **(B)** 10d, **(C)** 12d, **(D)** 14d. Phenotypic observation of WT and transgenic plant lines re-watered for **(E)** 7d, **(F)** 10d, **(G)** 12d, **(H)** 14d.

We examined the effects of drought stress on WT and transgenic poplar plants after growth in a greenhouse for 6 months. Under well-watered conditions, performance did not differ between the WT and the transgenic lines. However, after 7 days of drought stress, leaves of WT plants exhibited extensive dehydration symptoms, but only slight wilting was observed in OE14 and no effect was seen in OE18 (Figure 2A). After 14 days of drought stress, most of the WT leaves were chlorotic, while OE14 plant lines had less damage compared to WT plants, and most of the OE18 lines were green and vigorous (Figures 2B–D). After re-watering for 10 days, OE14 plants recovered faster than the WT plants (Figures 2E–H).

**FIGURE 3:**
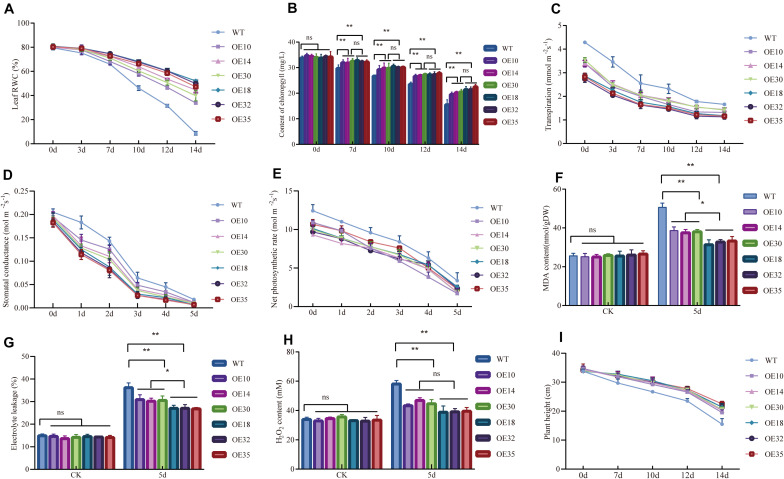
Physiological analysis of *LbDREB6* overexpression lines and WT plants under drought stress. Measurement of leaf relative water content (RWC) **(A)**, chlorophyll content **(B)**, the transpiration **(C)**, stomatal conductance **(D)**, net CO_2_ assimilation **(E)**, MDA content **(F)**, electrolyte leakage (EL) **(G)**, and H_2_O_2_ content **(H)**. **(I)** Plant height after being re-watered for 2 weeks. For **(I)**, each value represents the mean of 20 plants, and error bars represent ±SD. For **(A)** to **(H)**, data are presented as means of three biological replicates, and error bars represent ±SD. Asterisks indicate significant differences, *P < 0.05 and **P < 0.01.

During the drought period, the leaf RWC (%) showed a downward trend, especially for WT plants (Figure 3A). Furthermore, the chlorophyll level of the transgenic and WT plants also decreased, but the transgenic plants still had a higher chlorophyll content than WT plants (Figure 3B). Additionally, the transgenic and WT poplars had remarkable variations in transpiration rate (Tr), stomatal conductance (Gs), and net CO_2_ assimilation during the 5 days of drought stress. The Tr and Gs in the transgenic and WT poplars showed an overall decreasing trend but they decreased faster in WT plants than that in transgenic poplars after 2 days of drought stress (Figures 3C,D). The net CO_2_ assimilation in the transgenic and WT poplars both decreased, but the net CO_2_ assimilation in WT poplars decreased much more rapidly than that in the transgenic poplars after drought treatment (Figure 3E).

The MDA content (Figure 3F) and relative electrical conductance (EL) (Figure 3G) of WT and transgenic plants considerably increased after drought treatment. Among them, the MDA content (Figure 3F) and EL (Figure 3G) in transgenic plants decreased compared with WT plants. Meanwhile, MDA, and EL in high-level *LbDREB6* overexpression plants were less than those in moderate-level *LbDREB6* overexpression plants. The H_2_O_2_ content in the leaves of WT and transgenic plants was not significantly different compared to that before the treatment (Figure 3H). The H_2_O_2_ content in transgenic plant leaves was lower than that in WT plants after drought treatment, although both showed an increased H_2_O_2_ content (Figure 3H). Moreover, the H_2_O_2_ content was less in OE18, OE32, and OE35 compared to OE10, OE14, and OE30. After re-watering, the transgenic plants grew vigorously, and the height of OE14 was significantly higher than the WT plants (Figure 3I), while, WT plants were affected much more seriously and grew slowly (Figure 3I). We also verified the result in the flask, after 7% PEG6000 treatment for 2 days, the leaves of the WT (Supplementary Figure 2A) were damaged early and seriously, but the OE14 (Supplementary Figure 2B) and OE18 (Supplementary Figure 2C) plants still grew normally. This result was consistent with that of the experiment conducted in the greenhouse. Altogether, the transgenic poplars had better tolerance to drought stress than WT plants and high expression levels of *LbDREB6* with less damage suffered from drought stress.

### Expression Comparison of Downstream Target Genes Between OE14 and OE18 Leaf Transcriptomes in Response to Drought Stress

**FIGURE 4:**
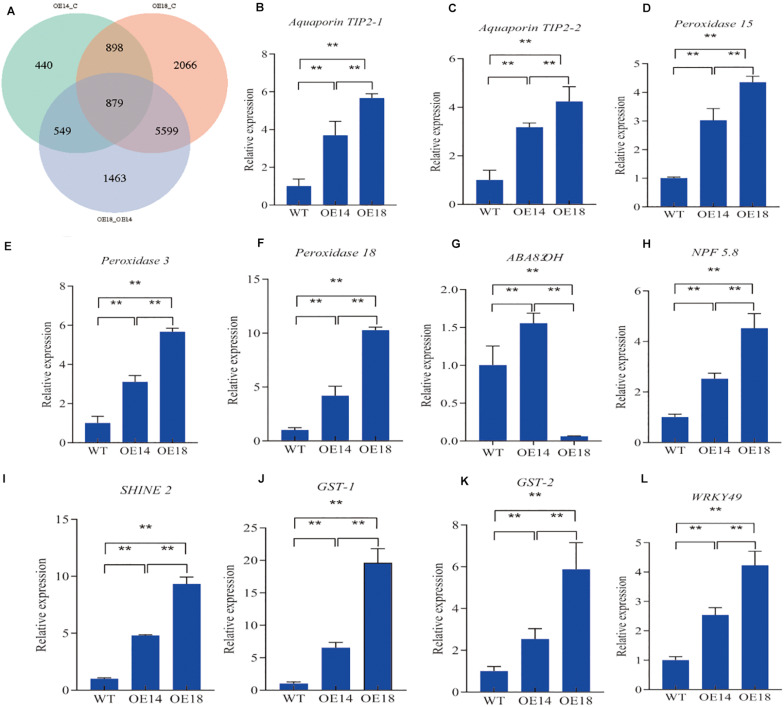
Quantitative real-time PCR analyses of the transcript levels of eight selected DEGs co-up-regulated in high-level (OE18) and moderate-level (OE14) *LbDREB6* overexpression lines compared with WT plants under drought stress for 5 days. **(A)** Venn diagram among WT and OE14, WT and OE18, and OE14 and OE18 plants. Quantitative real-time PCR analyses of the transcript levels of **(B)** *Aquaporin TIP2-1*, **(C)** *Aquaporin TIP2-2*, **(D)** *Peroxidase 15*, **(E)** *Peroxidase 3*, **(F)** *Peroxidase 18*, **(G)** *ABA8’OH*, **(H)** *NPF 5.8*, **(I)** *SHINE2*, **(J)** *GST-1*, **(K)** *GST-2*, **(L)** *WRKY49*. Data are presented as means of three biological replicates, and error bars represent ±SD. Asterisks indicate significant differences, *P < 0.05 and **P < 0.01.

We used the Illumina sequencing platform to sequence the leaf transcriptomes of the WT and *LbDREB6*-transgenic plants under drought stress for 7 days. We identified a total of 20698 differentially expressed genes (DEGs) during drought stress (*P* < 0.05 and FDR < 0.001). Among these DEGs, there were 2,766 (1,654 up, 1,112 down, Supplementary Data 1) in OE14 and 9,442 (5,862 up, 3,580 down, Supplementary Data 2) in OE18 compared to the WT, respectively, and 8,490 (5,213 up, 3,277 down, Supplementary Data 3) were identified in OE18 compared to OE14 plants. Finally, a total of 879 DEGs were common in three groups (Supplementary Data 4 and Figure 4A). Of the common 879 DEGs, there were 707 up-regulated and 172 down-regulated genes; we found that multiple drought stress related downstream genes were differentially expressed, such as aquaporin (AQP) tonoplast intrinsic protein (TIP), glutathione S-transferase (*GST*), and some transcriptional factors. To verify the NGS data, the expression levels of eight genes were examined in WT, OE14, and OE18 plants with drought treatment by qRT-PCR (Figures 4B–H). It was found that the expression levels of all the genes tested were increased in the transgenic plants compared to the WT plants under drought treatment except *ABA8’OH* and *WRKY46* in OE18 (Figure 4G). Among them, the expression levels of seven genes were higher in OE18 than those in OE14 plants (Figures 4B–F,H–L), and there were no significant differences in expression levels of the five genes between OE14 and OE18 plants (Supplementary Figures 4A–E) except WRKY46 (Supplementary Figure 4F). It was likely that these DEGs resulted in drought resistance of OE18 and OE14 when compared to WT plants. In OE18, we also found some DEGs that were probably related to the drought tolerance phenotype when compared to OE14 plants (Supplementary Data 3). Most of these DEGs were up-regulated, for example, major intrinsic protein (*MIP*) (Potri.003G050900, Potri.005G109300, and Potri.006G121700), cellulose synthase-like protein D5 (*CSLD5*) (Potri.014G125100), 2 galactinol synthase genes (*GolS*) (Potri.002G191600 and Potri.010G150400), and 5 late embryogenesis abundant (*LEA*) protein genes (Potri.018G052500, Potri.002G124600, Potri.009G003800, Potri.010G012100, and Potri.010G012100) responded to water deprivation; 2 peroxidase genes (Potri.007G122100 and Potri.018G015500), thioredoxin gene (Potri.001G028500), peptide methionine sulfoxide reductase B5 (*MSRB5*) (Potri.008G198600), L-ascorbate oxidase (Potri.004G010100), and peptide methionine sulfoxide reductase (MSRA) (Potri.T135400) respond to oxidative stress; 3 *ABP19a* (Potri.013G141900, Potri.001G169000, and Potri.003G065300), Metalloendoproteinase 2-MMP (Potri.019G073800), and 2 *GST* (Potri.011G140400 and Potri.011G140600) genes were involved in auxin-activated signaling pathways and development processes. We randomly selected some related genes to conduct qRT-PCR verification. The qRT-PCR analysis result showed that *ABP19a* (Potri.001G169000), *TIP2-1* (Potri.003G050900), *PIP2-8* (Potri.005G109300), *LEA* (Potri.018G052500), and *Peroxidase 18* (Potri.018G015500) were highly induced in OE18 compared to OE14 except *GolS* (Potri.010G15040) (Supplementary Figure 5). These results were consistent with the transcriptome data.

### Effect of High- and Moderate-Level LbDREB6 Overexpression on Susceptibility to Marssonina Brunnea

**FIGURE 5:**
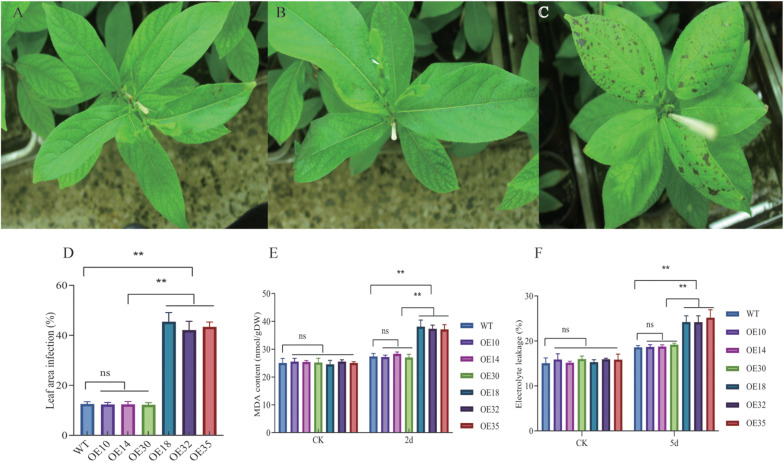
Comparison of *LbDREB6* overexpression lines and WT leaves after *Marssonina brunnea* infection. The WT **(A)**, OE14 **(B)**, and OE18 **(C)** plants after being infected with *M. brunnea* for 2 days. Bar = 2cm. **(D)** Measurement of leaf disease indices. Each value represents the mean of 20 plants, and error bars represent ±SD. Measurement of MDA content **(E)** and electrolyte leakage (EL) **(F)** after being infected with *M. brunnea* for 2 days. Data are presented as means of three biological replicates, and error bars represent ±SD. Asterisks indicate significant differences, *P < 0.05 and **P < 0.01.

After inoculation with the fungus *M. brunnea*, the OE14 line (Figures 5B,D) displayed no significant difference in phenotype compared to the WT (Figures 5A,D), while the lesion area on OE18 was larger than that of the WT (Figures 5C,D). This suggests that the enhanced expression level of *LbDREB6* affects the susceptibility of poplar to the fungus. The MDA content of WT, OE14, and OE18 lines considerably increased after pathogen treatment (Figure 5E); the MDA content in OE18 plants was greater than that in WT plants compared to OE14 plants (Figure 5E). In addition, the relative EL showed the same trend of change (Figure 5F), indicating the leaves of OE18 plants showed severe membrane damage compared to OE14 plants. Overall, the susceptibility to *M. brunnea* infection in OE18 increased and that in OE14 was not altered compared to WT plants.

### Comparison of OE18 and OE14 on Expression of Genes Involved in Disease Tolerance Under Pathogen Infection

To determine the possible pathways of LbDREB6 in pathogen defense, we conducted transcriptomic sequencing of leaf inoculated with the pathogen *M. brunnea*, for two days, from which we obtained 53.20 Gb clean reads (18.2–24.7 million per library, Q30 ≥ 95.02%). Approximately 68.3–70.42% clean reads per library could be mapped to the *P. trichocarpa* genome (Supplementary Table 3). The quality of the assembled transcriptome was appropriate for functional annotation and further analysis compared with the WT.

**FIGURE 6:**
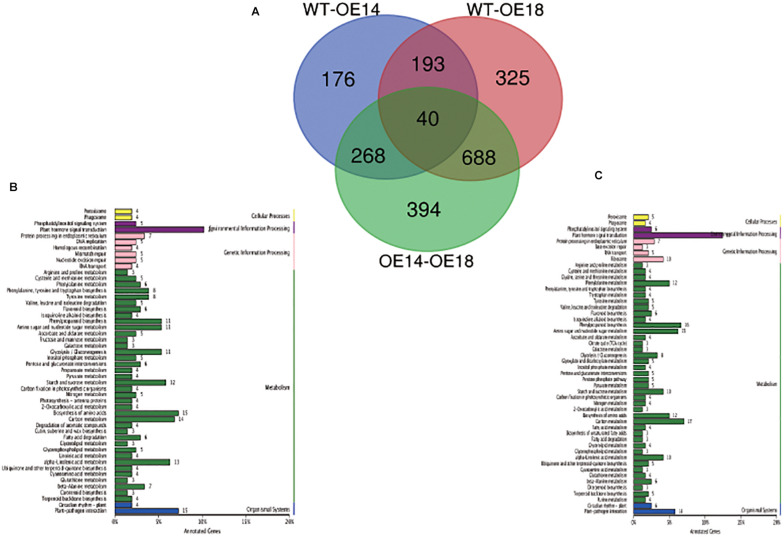
Venn diagram and KEGG classification of the DEGs among high-level (OE18) and moderate-level (OE14) *LbDREB6* overexpression lines and WT *P. ussuriensis* plants after infection with *Marssonina brunnea*. **(A)** Venn diagram among WT and OE14, WT and OE18, and OE14, and OE18 plants. **(B)** KEGG classification of differentially expressed genes between WT and OE18 lines. **(C)** KEGG classification of differentially expressed genes between OE14 and OE18 lines.

Based on the transcriptome annotation, a total of 688 DEGs were identified in OE18 when compared to both the WT and OE14 plants (Figure 6A and Supplementary Data 3). In total, 58 DEGs related to disease tolerance were identified in OE18 plants including 41 up-regulated and 17 down-regulated genes (Supplementary Data 3). Plants have two different plant–pathogen interaction sub-pathways, pattern triggered immunity (PTI) and effector-triggered immunity (ETI) (He et al., 2015). In PTI, there were 17 genes differently expressed in OE18 when compared to both the WT and OE14 plants, including translated nucleotide-binding leucine-rich repeat (NB-LRR) kinase EFR, two WRKY TFs (WRKY49 and WRKY7), three ubiquitin E3 ligases, and some receptor-like proteins involved in immune response and regulating the expression downstream defense-related genes. In ETI, we found that two disease tolerance proteins RPM1 and one disease tolerance protein RPS2 were down-regulated. In addition, two nitrite reductase differentially induced genes were down-regulated in OE18 when compared to both the WT and OE14 plants. KEGG enrichment revealed that DEGs related to plant hormone signal transduction, followed by plant–pathogen interactions and biosynthesis of amino acids were enriched in OE18 plants when compared to both the WT and OE14 plants (Figure 6B); similarly, plant hormone signal transduction and plant–pathogen interactions were enriched in OE18 plants compared with both WT and OE14 plants (Figure 6C). In plant hormone pathways, one PYR/PYL family protein, two protein phosphatases 2C (PP2C), and two auxin-binding proteins ABP19a were also differentially expressed.

**FIGURE 7:**
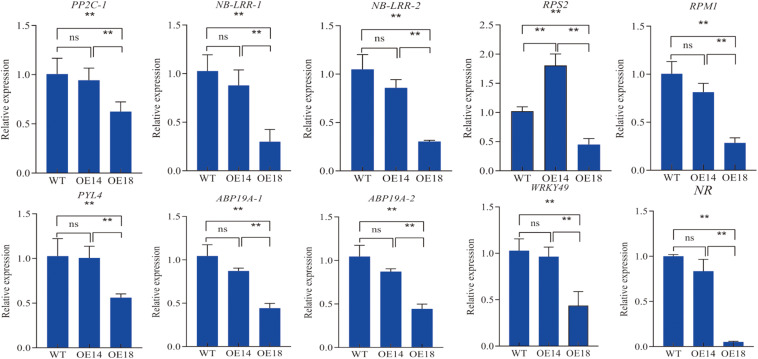
Quantitative real-time PCR analyses of the transcript levels of disease tolerance related genes in leaves of high-level (OE18) and moderate-level (OE14) *LbDREB6* overexpression lines and WT *P. ussuriensis* plants under *M. brunnea* infection treatment. Data are presented as means of three biological replicates, and error bars represent ±SD. Asterisks indicate significant differences, *P < 0.05 and **P < 0.01.

We selected some DEGs involved in plant–pathogen interaction pathways and ABA regulation to conduct qRT-PCR analysis. The result showed *PP2C-1, NB-LRR-1, NB-LRR-2, RPS2, RPM1-1, RPM1-2, PYL4, WRKY49*, and *NR* genes were down-regulated in OE18 plants when compared to both the WT and OE14 plants (Figure 7).

### Effect of High-Level and Moderate-Level LbDREB6 Overexpression on GA20ox1 Gene Expression

**FIGURE 8:**
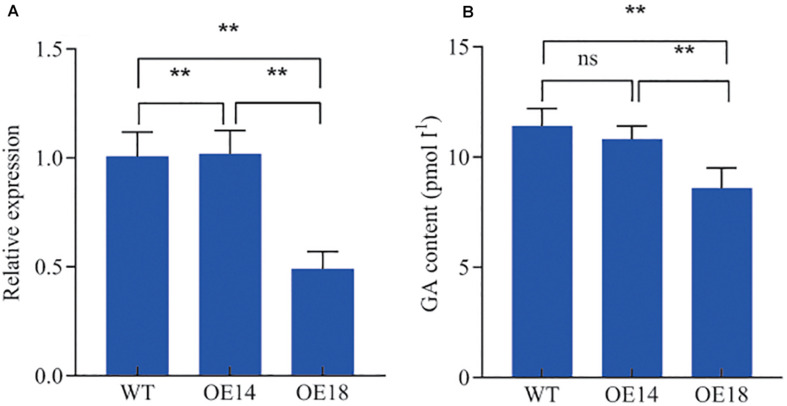
Quantitative real-time PCR analyses of the transcript levels of the *GA20ox1* gene and GA content detection in leaves of high-level (OE18) and moderate-level (OE14) *LbDREB6* overexpression lines and WT *P. ussuriensis* plants. **(A)** qRT-PCR analysis of the *GA20ox1* gene. **(B)** GA content determination in leaves of WT and transgenic plants. Data are presented as means of three biological replicates, and error bars represent ±SD. Asterisks indicate significant differences, *P < 0.05 and **P < 0.01.

We found that the *GA 20-oxidase 1* (*GA20ox1*) gene was down-regulated in OE18 plants when compared to both WT and OE14 plants from the shoot tip transcriptome dataset. The qRT-PCR results showed that the expression level of *GA20ox1* in OE18 was decreased to ∼50 and 49% when compared to the WT plants and OE14, respectively (Figure 8A). However, the expression of *GA20ox1* was not significantly different between the OE14 and WT plants (Figure 8A). To investigate if there is a difference in GA content between WT and transgenic plants, all samples of shoot tips were harvested after plants were transplanted into pots for 2 months. There was no significant difference between the WT or moderate-level *LbDREB6* overexpression plants, but GA in high-level *LbDREB6* overexpression plants was significantly lower, by ∼25%, when compared to the WT plants (Figure 8B). This was consistent with the gene expression pattern of *GA20ox1* in the samples (Figure 8A). To examine whether the dwarf phenotype was caused by GA deficiency, we cultivated WT and transgenic plantlets transplanted in a greenhouse under natural sunlight for 2 months and they were sprayed daily with 100 μM GA_3_ for 15 days. Under these conditions, the height of the transgenic as well as the WT plants increased rapidly, especially for OE18 plants (Supplementary Figure 3). The increased ratio of OE18 plant height was higher than that of WT plants (Supplementary Figure 3). These results demonstrated that high-level overexpression of *LbDREB6* decreased growth rate, probably by inhibiting GA synthesis, while moderate overexpression of *LbDREB6* did not change the normal growth of transgenic *P. ussuriensis*.

## Discussion

Increasingly, numerous studies are showing that DREBs have crucial roles in regulating plant development and responses to both abiotic and biotic stresses. However, there have been only a few studies to date to investigate the multi-directional effects of DREBs in trees. In the present study, we overexpressed *LbDREB6* in poplar to investigate the role of this A-6 DREB TF in regulating plant growth, drought tolerance, and disease tolerance. We found that in the transgenic line of poplar with high levels of overexpression of *LbDREB6*, drought tolerance was greatly improved, but growth was inhibited, and there was a decreased tolerance to fungal pathogens. In contrast, in the plants having a moderate level of overexpression of *LbDREB6*, we observed drought tolerance along with normal growth and no effects on pathogen tolerance. Our study suggests that drought tolerance can be improved by carefully adjusting the levels of overexpression of DREB TF genes to avoid the occurrence of unfavorable effects.

The abilities of plants to respond to drought stress can sometimes be predicted by their varying capacities to modulate key physiological and biochemical responses at the cellular level, including changes in membrane integrity, internal water balance, and the accumulation of osmolytes and antioxidants (Kang et al., 2011). MDA, the final product of lipid-peroxidation, is one well-known marker of cell membrane injury (Taulavuori et al., 2001; Anjum et al., 2015), while accumulation of reactive oxygen species (ROS) can be scavenged by antioxidants (Royer and Noctor, 2003). Our results showed that the levels of MDA, H_2_O_2_, and ROS, in plants overexpressing *LbDREB6* under water deficit conditions were lower compared to WT plants. The lower levels of H_2_O_2_ may indicate that *LbDREB6* conferred enhanced protection from oxidative damage and a better ROS scavenging ability in the transgenic plants. Lower MDA levels seem to indicate that the transgenic plants had greater membrane integrity to withstand cellular-level effects of water loss. Our results were similar to observations in *35S:MsDREB6.2*-transgenic apple, which had improved tolerance to drought stress (Liao et al., 2017) and coincided with lower levels of ROS and MDA. Another measure of membrane integrity is EL (Gu et al., 2017). In this study, the lower rate of increase in EL in plants overexpressing *LbDREB6* suggests that less cell damage occurred from drought stress. This physiological change within the overexpression lines may account for the higher RWC compared to WT plants under drought stress. These results indicate that the *LbDREB6*-overexpressing plants achieved better water balance to alleviate water deficiency and had higher drought tolerance compared to the non-transgenic WT plants. Additionally, both OE14 and OE18 showed better growth compared to WT plants under drought stress, though the OE18 line exhibited greater drought tolerance than OE14.

When the plants were subjected to drought stress, a large number of genes were differentially expressed compared to plants under normal growth conditions, and subsequently, many protein or metabolic products were generated to help protect the plant from drought stress. In this study, overexpression of *LbDREB6* yielded increased transcription of many downstream genes. This is expected because TFs like *LbDREB6* are significant upstream regulatory proteins, which play a major role in multiple pathways when plants are subjected to drought stress (Ning et al., 2011). Prior studies also revealed that transcription of downstream genes was increased when DREB TFs were up-regulated. For example, *MdSHINE2* from apple (a homolog of *AtSHINE2* in *Arabidopsis*), an A-6 DREB, conferred drought tolerance by regulating wax biosynthesis (Zhang et al., 2019). Similarly, *Arabidopsis* transformed with *GmWRKY54*, an A-6 DREB from soybean, conferred salt and drought tolerance, possibly through the regulation of *DREB2A* and *STZ/Zat10* (Zhou et al., 2008). Additionally, *RAP2.4* in *Arabidopsis* regulated the expression of the plasma membrane intrinsic protein (PIP) and TIP subfamilies of AQPs, which are integral membrane proteins, in response to drought stress (Rae et al., 2011). AQPs, especially PIP and TIP, are believed to play key roles in maintaining water homeostasis (Alexandersson et al., 2005; Rae et al., 2011).

In the current study, we found differential expression of several genes involved in drought response pathways between the transgenic lines and the WT. Notably, we found that two *GST* genes, which constitute part of an antioxidant defense system, were induced in transgenic *LbDREB6* overexpression lines. Activation of the GST antioxidant system occurred in response to ROS (Noctor et al., 2014; Fox et al., 2017) and indicates that the poplar trees were protected from ROS that resulted from drought. We also found that a key regulator of ABA catabolism, *ABA8’OH* (Potri.004G235400), was down-regulated in the OE18 line when compared to OE14. ABA is an important phytohormone that regulates plant water use, and thus, it is correlated with drought tolerance. While ABA can be applied exogenously to facilitate drought tolerance, this approach has limited utility because ABA is rapidly inactivated. Therefore, genes that regulate levels of endogenous ABA are of critical importance to develop drought resistant crops (Takeuchi et al., 2016). Down-regulation of *ABA8’OH* should decrease catabolism of ABA, and therefore probably improves drought tolerance in the OE18 line. Taken together, these results suggest that *LbDREB6* can regulate large groups of stress-related genes, and subsequently, promote enhanced tolerance against drought stress.

Other genes known for their involvement in drought response, such as *MIP*, *TIP*, *GolS*, and *LEA*, showed a greater change in expression levels in the OE18 plants compared to OE14. *GolS* of *Arabidopsis*, *GolSAt2*, has been reported to confer drought tolerance and increase grain yield in transgenic rice (*Oryza sativa* L.) under dry field conditions (Selvaraj et al., 2017). *LEA* of wheat, *TaLEA3*, overexpressed in *Phellodendrons amurense* yielded tolerance to drought stress by rapid stomatal closure (Yang et al., 2018). Differential expression of homologs of these genes in OE18 may help to explain why this line is more drought resistant than OE14 at the transcriptional level.

Gibberellic acid, the most important hormone regulating shoot elongation, plays an important role in determining plant height (Richards et al., 2001). One critical gene family that participates in GA synthesis and degradation is *GA20ox*, an oxidase (Hedden and Phillips, 2000; Wuddineh et al., 2015). Decreasing the level of expression of *GA20ox* causes severe dwarf phenotypes in some species due to reduced levels of active GAs. In tomato, *SlDREB* is known to act as a positive regulator in drought stress responses, but overexpression caused the dwarf phenotype via down-regulation of *GA20oxs* as well as ent-copalyl diphosphate synthase (*SlCPS*), another gene in the GA synthesis pathway (Li et al., 2012). The dwarf phenotype also affected *DREB1B/CBF1* transgenic tomato, which could be rescued by exogenous application of GA_3_ (Hsieh et al., 2002). The expression levels of GA biosynthetic genes, including *OsGA20ox1*, were significantly reduced in rice plants overexpressing *JcDREB2* of *Jatropha curcas* Tang et al., 2017. Similarly, here, we found that the expression level of *GA20ox1* in OE18 was decreased by ∼50% when compared to WT and OE14 plant lines, and OE18 showed lower GA content. The expression level of *GA20ox1* was not significantly different between the WT and OE14, which were also similar in height. Therefore, it appears that dwarfism due to overexpression of *LbDREB6* results from an interaction that down-regulates expression of the *GA20ox* gene family, and consequently, reduces the levels of GAs.

The poplar rust fungus, *Marssonina brunnea* (Ellis and Everh.) Magnus causes significant yield reduction and severe economic losses in commercial poplar plantations. In our study, OE18 was more sensitive to *M. brunnea* infection compared to both OE14 and WT plants, while we detected no difference between OE14 and WT plants. This suggests that tolerance to fungal infection was reduced by overexpression of *LbDREB6*. Briefly, plants have two tiers of responses to microbial and fungal pathogens: pattern triggered immunity (PTI) at epidermal layers in response to the pathogens themselves and effector triggered immunity (ETI), which responds to metabolites produced by pathogens after an infection is established (Boller and He, 2009). Both PTI and ETI recognize pathogen infection according to arrays of receptors that include kinases, TFs, and plant hormones (He et al., 2015). Additionally, pathogenic response in plants is mediated by plant hormones, such as salicylic acid, jasmonic acid, and ABA, which act as secondary messengers (Mauch-Mani and Mauch, 2005; He et al., 2015). We found that multiple pattern recognition receptors that participate in the PTI system, especially EF-Tu receptors (ETRs), were differentially expressed in the OE18 line, in which they were largely down-regulated compared to OE14 and WT plants. In particular, we observed that the ETRs, WRKY (WRKY49 and WRKY7), *RPM1, RPS2*, and *NOS* were down-regulated in OE18. WRKY TFs are known to play important roles in transcriptional reprogramming in response to various stressors including pathogen infection in plants. For example, in rice, *OsWRKY67* improved tolerance against two pathogens, *Magnaporthe oryzae* (T. T. Hebert) M. E. Barr and *Xanthomonas oryzae oryzae* (Vo et al., 2018). *NOS* genes regulate the production of nitric oxide, which is involved in the plant pathogenic response (Arasimowicz-Jelonek and Floryszak-Wieczorek, 2014). Thus, down-regulation of *NOS* in OE18 likely led to reduced nitric oxide, and therefore, decreased pathogen tolerance. Additionally, several genes affecting levels of ABA were also down-regulated in OE18. Specifically, we found that ABA receptors, including one *PYL4* and one *PP2C*, were down-regulated in OE18 as well as ABA responsive element binding factor (ABF), which encodes a basic leucine zipper TF. The PYR/PYL family of proteins positively regulate ABA response in various tissues by inhibiting the interaction of PP2C with a protein kinase, SnRK2 (Umezawa et al., 2009). Then, this complex stimulates the expression of ABF, which enhances tolerance to necrotrophic pathogens (Umezawa et al., 2009). Thus, we proposed that high levels of overexpression of *LbDREB6* yielded decreased tolerance to *M*. *brunnea* due to effects on the PTI system and actions of ABA. However, this behavior of *LbDREB6* merits further study.

## Conclusion

In previous studies, the overexpression of DREBs in transgenic plants improved drought tolerance but with varying levels of growth inhibition under non-drought conditions. Our results show that the higher the overexpression level of the *LbDREB6* gene, the stronger the drought tolerance in *Populus ussuriensis*. However, under high levels of overexpression of *LbDREB6*, poplar trees exhibited growth inhibition and decreased tolerance to fungal infection. In contrast, under moderate levels of overexpression *LbDREB6*, poplar trees showed normal growth and no effect on fungal tolerance. These results provide novel insight into the regulation of plant growth by *LbDREB6* and its roles in diverse responses to biotic and abiotic stressors. Additionally, our study provides a method to achieve improved drought tolerance by adjusting the levels of overexpression of *DREB* genes to avoid the occurrence of unfavorable traits, such as decreased growth rate and reduced disease tolerance.

## Data Availability Statement

Publicly available datasets were analyzed in this study. This data can be found here: GSE139373 and GSE120118.

## Author Contributions

CL designed and conceived the experiments. JY and HW performed the experiments. SZ and XL analyzed the data. XZ and WW revised the manuscript. CL and JY wrote the manuscript. All authors reviewed and approved the manuscript.

## Conflict of Interest

The authors declare that the research was conducted in the absence of any commercial or financial relationships that could be construed as a potential conflict of interest.

## Footnotes

1 http://bioinfo.cau.edu.cn/agriGO/analysis.php

2 http://kobas.cbi.pku.edu.cn/program.run.do

## Supplementary Material

Supplementary Figure 1 Generation and transplantation of *LbDREB6* transgenic *Populus ussuriensis* lines. **(A)** PCR validations on OE lines; M, DNA Marker DL2000; P, pROKII-GFP plasmid used as the positive control, N, gDNA of the wild type (WT) plant was utilized as the PCR templates for the negative control; lanes 4–12, gDNA of *LbDREB6* overexpression lines were utilized as the PCR templates for lane 4–7. **(B)** Transplantation of *in vitro* plants with well-developed leaf and root systems in pots containing autoclaved sand and soil mixture (1:3 v/v) for 3 months. Left: WT, middle: OE14 line, right: OE18 line. **(C)** Detection of the LbDREB6 protein in transformed *P. ussuriensis* by Western blotting.

Supplementary Figure 2 Exogenous GA 3 effectively reverses the GA-deficiency phenotype. Data are presented as means of three biological replicates, and error bars represent ±SD. Asterisks indicate significant differences, ^∗^*P* < 0.05 and ^∗∗^*P* < 0.01.

Supplementary Figure 3 Quantitative real-time PCR analyses of the transcript levels of eight selected DEGs co-up-regulated in high-level (OE18) and moderate-level (OE14) *LbDREB6* overexpression lines compared to WT plants under drought stress for 5 days. **(A)** *DREB2C*. **(B)** *Peroxidase 6*. **(C)** *ATHB-13*. **(D)** *MFYA.* **(E)** *WRKY40*. **(F)** *WRKY46*. Data are presented as means of three biological replicates, and error bars represent ±SD. Asterisks indicate significant differences, ^∗^*P* < 0.05 and ^∗∗^*P* < 0.01.

Supplementary Figure 4 Quantitative real-time PCR analyses of the transcript levels of eight selected DEGs co-up-regulated in in high-level (OE18) and moderate-level (OE14) *LbDREB6* overexpression lines compared to WT plants under drought stress for 5 days. Data are presented as means of three biological replicates, and error bars represent ±SD. Asterisks indicate significant differences, ^∗^*P* < 0.05 and ^∗∗^*P* < 0.01.

Supplementary Table 1 Primers used in qRT-PCR analysis for genes responsive to drought.

Supplementary Table 2 Primers used in qRT-PCR analysis for disease tolerance related genes.

Supplementary Table 3 Statistical analysis of sequencing.

Supplementary Data 1 The differentially expressed genes in OE14 compared to the WT plant.

Supplementary Data 2 The differentially expressed genes in OE18 compared to the WT plant.

Supplementary Data 3 The differentially expressed genes in OE18 compared to the OE14 plant.

Supplementary Data 4 Genes related to disease tolerance in OE18 line compared to the WT and OE14 lines.
