# Supplementary material for: Overexpression Levels of LbDREB6 Differentially Affect Growth, Drought, and Disease Tolerance in Poplar
Source: Front Plant Sci. 2020 Nov 11;11:528550. doi: 10.3389/fpls.2020.528550 (PMC7693672; doi:10.3389/fpls.2020.528550)
Supplement: Supplementary Figure 1 — Generation and transplantation of LbDREB6 transgenic Populus ussuriensis lines. (A) PCR validations on OE lines; M, DNA Marker DL2000; P, pROKII-GFP plasmid used as the positive control, N, gDNA of the wild type (WT) plant was utilized as the PCR templates for the negative control; lanes 4–12, gDNA of LbDREB6 overexpression lines were utilized as the PCR templates for lane 4–7. (B) Transplantation of in vitro plants with well-developed leaf and root systems in pots containing autoclaved sand and soil mixture (1:3 v/v) for 3 months. Left: WT, middle: OE14 line, right: OE18 line. (C) Detection of the LbDREB6 protein in transformed P. ussuriensis by Western blotting. [file Presentation_1.PPT]

## Slide 1
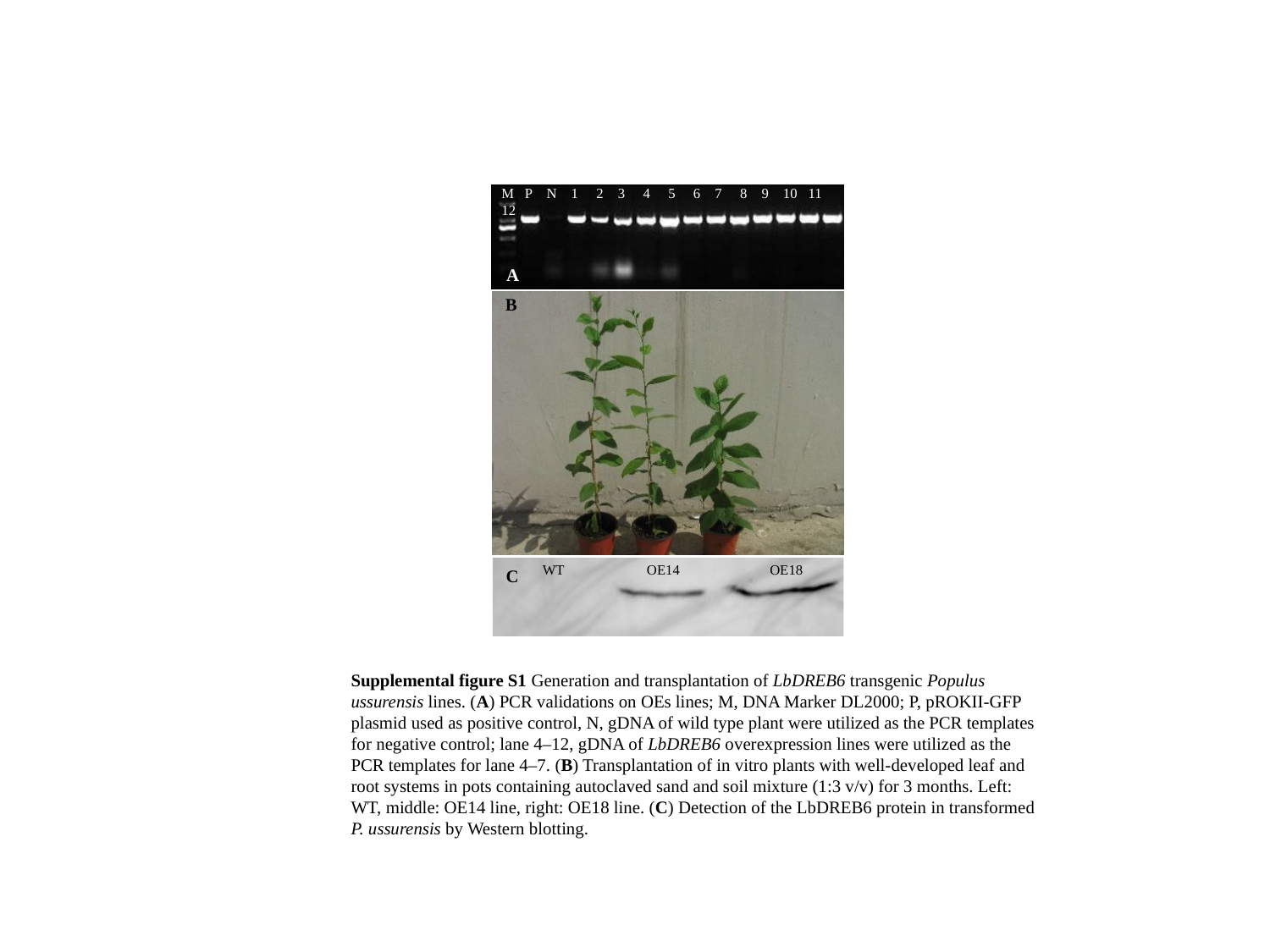

M P N 1 2 3 4 5 6 7 8 9 10 11 12
A
B
WT OE14 OE18
C
Supplemental figure S1 Generation and transplantation of LbDREB6 transgenic Populus ussurensis lines. (A) PCR validations on OEs lines; M, DNA Marker DL2000; P, pROKII-GFP plasmid used as positive control, N, gDNA of wild type plant were utilized as the PCR templates for negative control; lane 4–12, gDNA of LbDREB6 overexpression lines were utilized as the PCR templates for lane 4–7. (B) Transplantation of in vitro plants with well-developed leaf and root systems in pots containing autoclaved sand and soil mixture (1:3 v/v) for 3 months. Left: WT, middle: OE14 line, right: OE18 line. (C) Detection of the LbDREB6 protein in transformed P. ussurensis by Western blotting.

## Slide 2
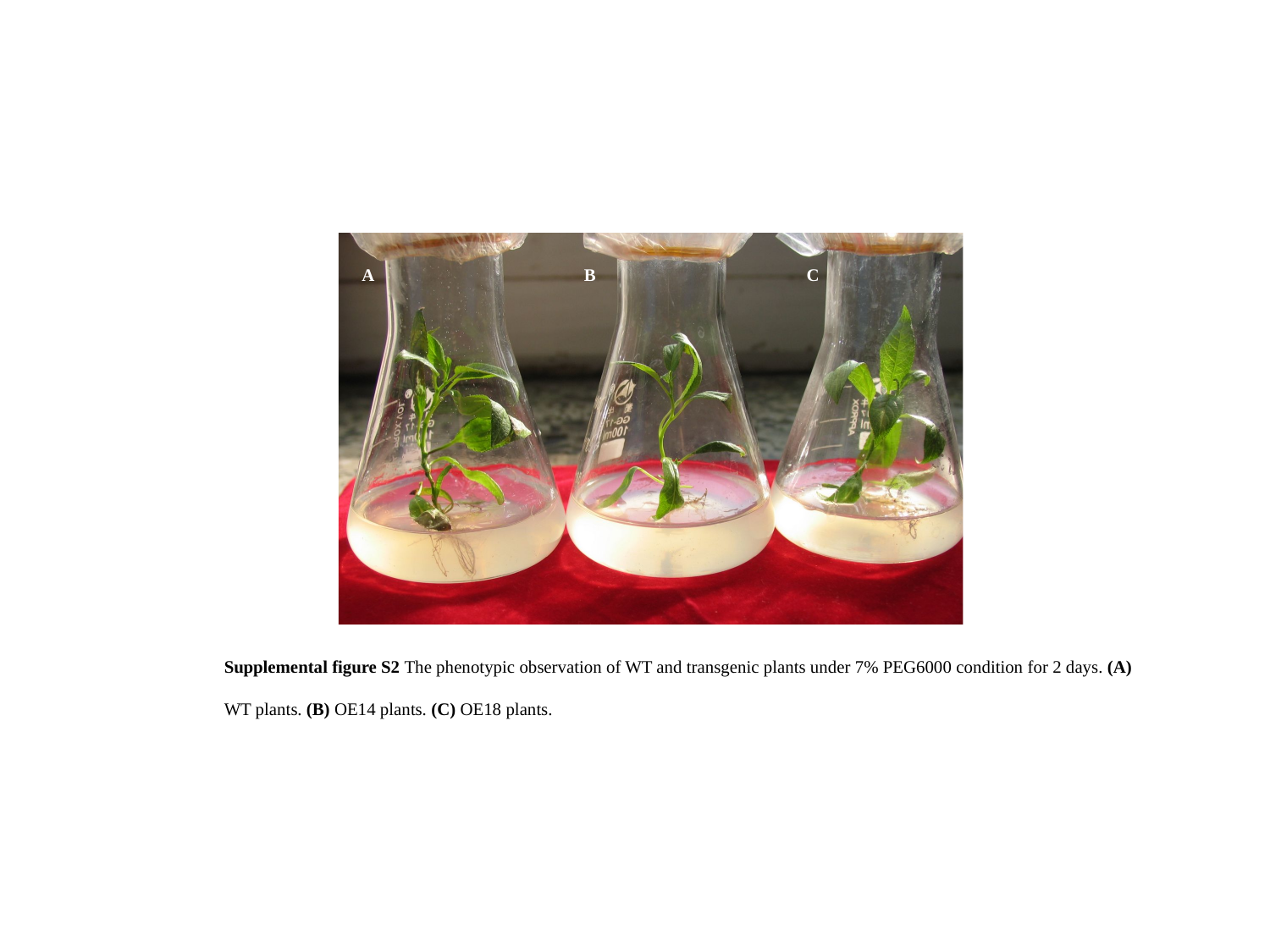

A
B
C
Supplemental figure S2 The phenotypic observation of WT and transgenic plants under 7% PEG6000 condition for 2 days. (A) WT plants. (B) OE14 plants. (C) OE18 plants.

## Slide 3
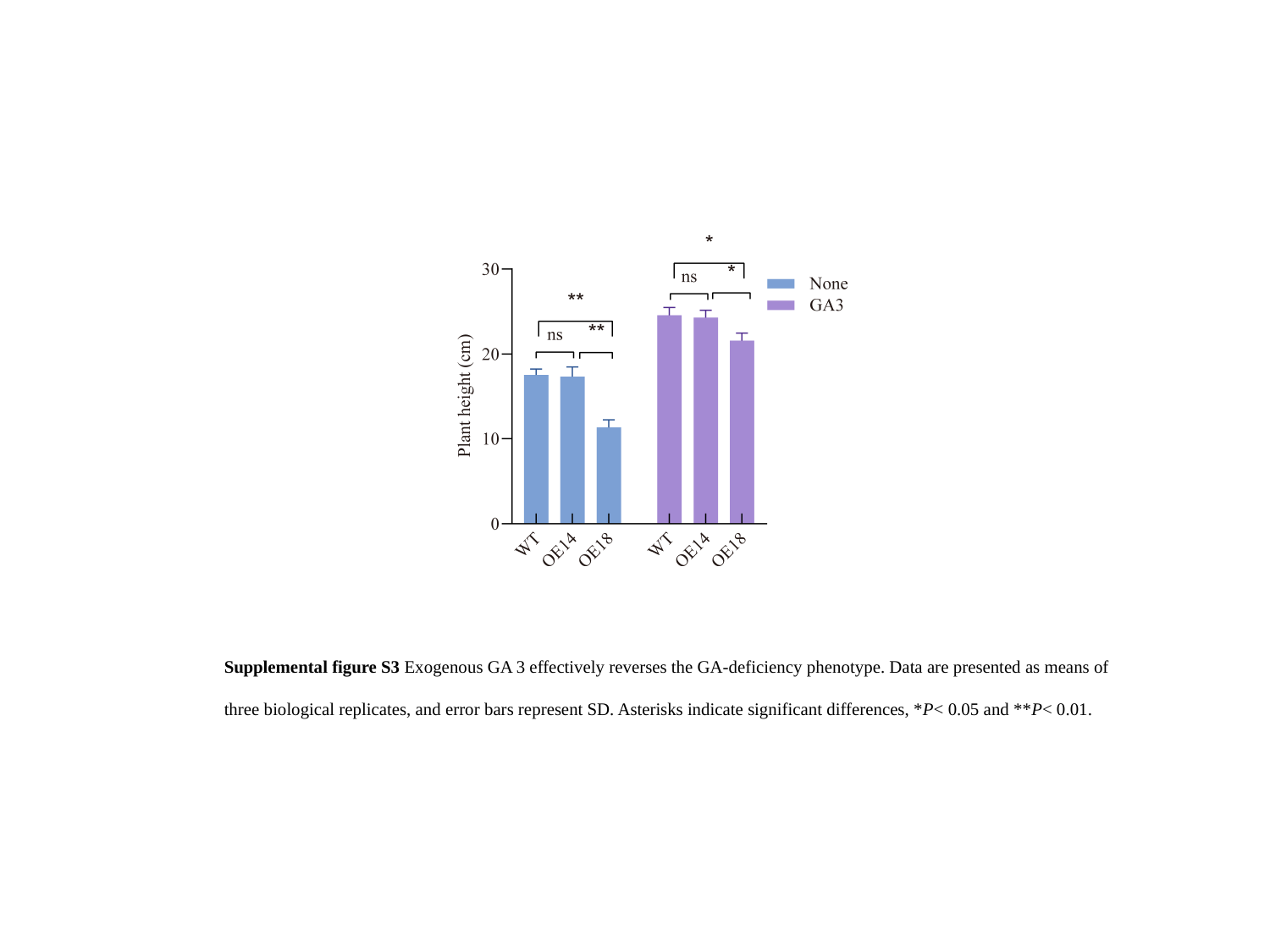

Supplemental figure S3 Exogenous GA 3 effectively reverses the GA-deficiency phenotype. Data are presented as means of three biological replicates, and error bars represent SD. Asterisks indicate significant differences, *P< 0.05 and **P< 0.01.

## Slide 4
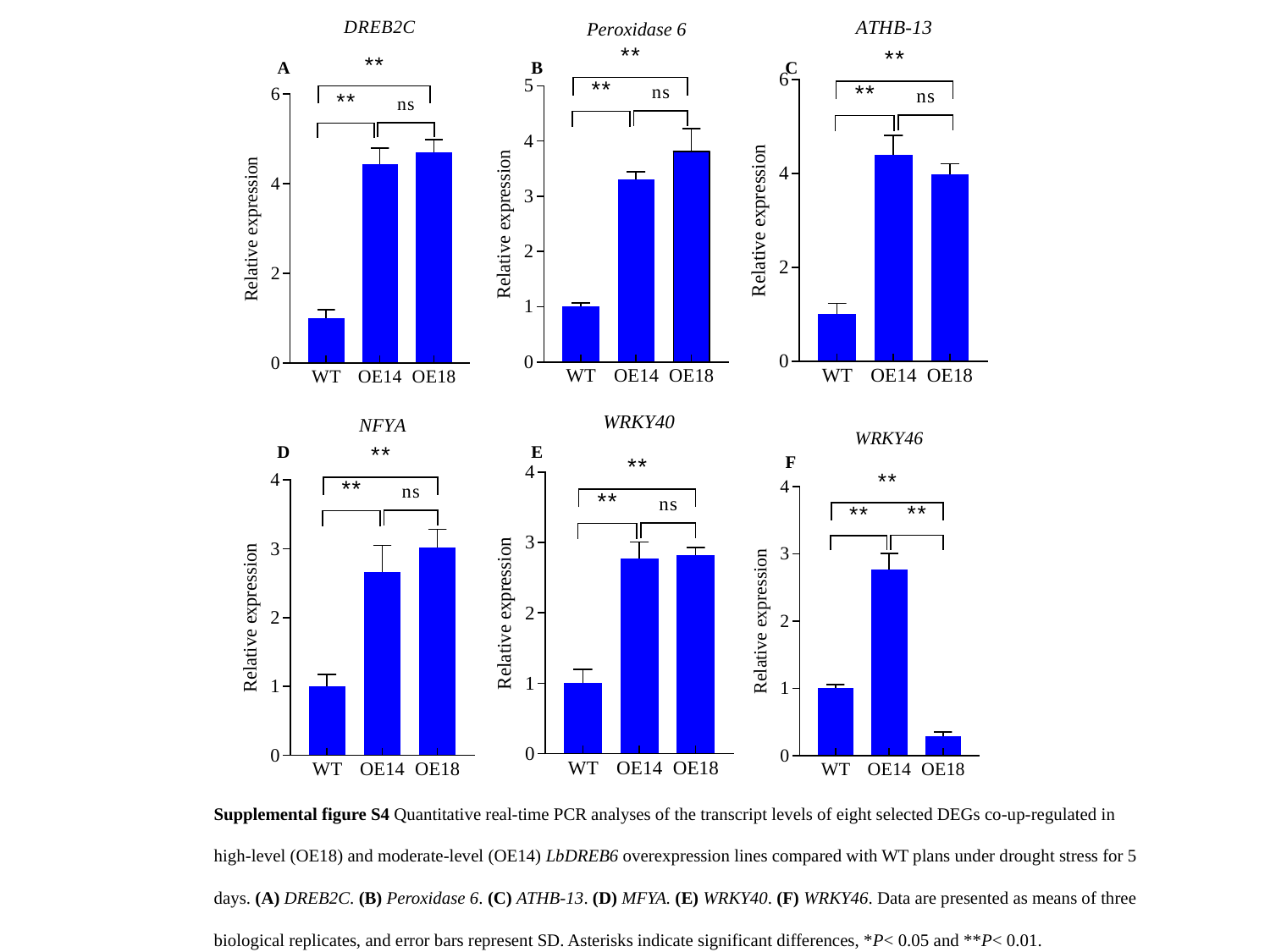

A
B
C
D
E
F
Supplemental figure S4 Quantitative real-time PCR analyses of the transcript levels of eight selected DEGs co-up-regulated in high-level (OE18) and moderate-level (OE14) LbDREB6 overexpression lines compared with WT plans under drought stress for 5 days. (A) DREB2C. (B) Peroxidase 6. (C) ATHB-13. (D) MFYA. (E) WRKY40. (F) WRKY46. Data are presented as means of three biological replicates, and error bars represent SD. Asterisks indicate significant differences, *P< 0.05 and **P< 0.01.

## Slide 5
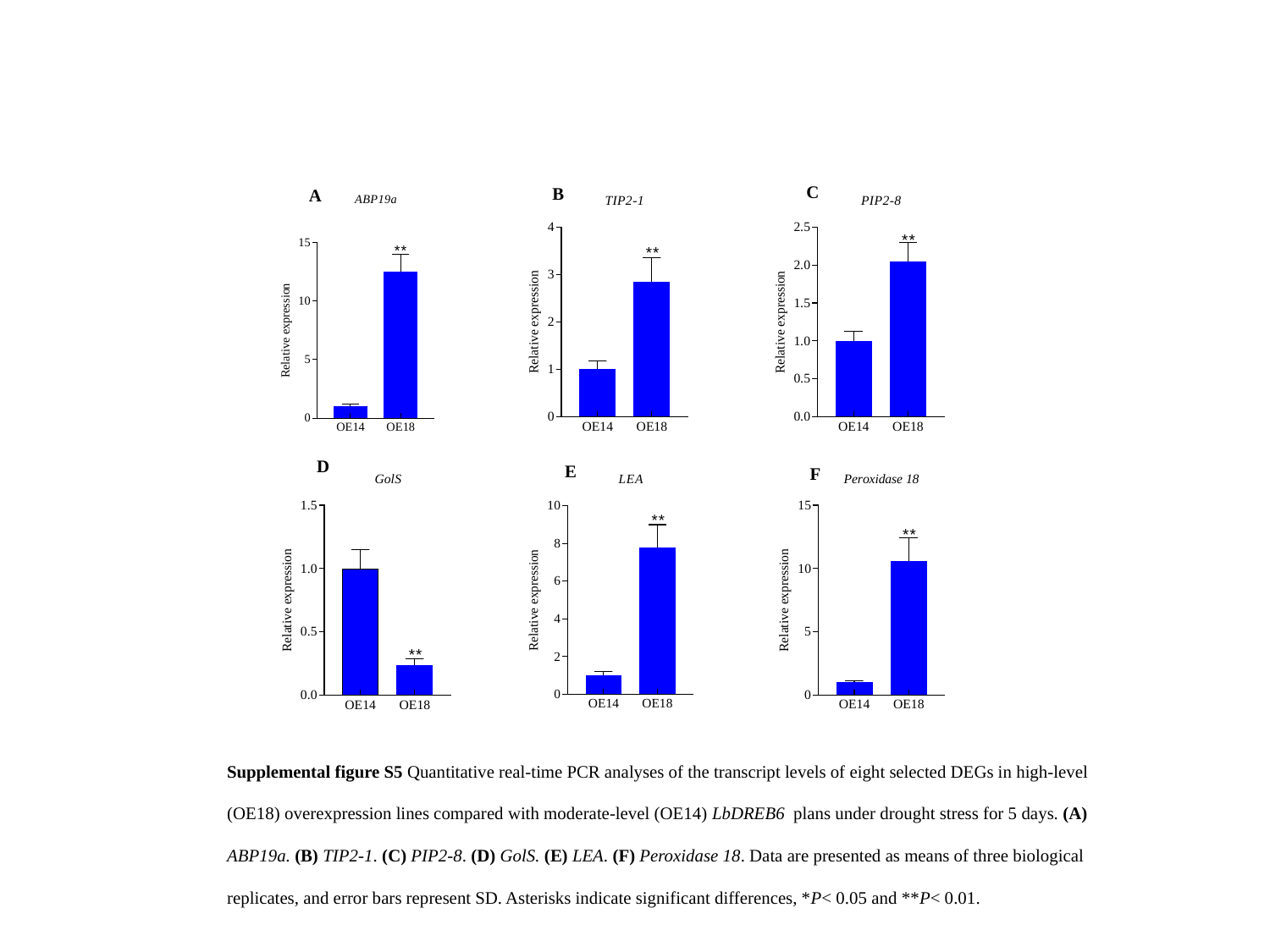

C
B
A
D
E
F
Supplemental figure S5 Quantitative real-time PCR analyses of the transcript levels of eight selected DEGs in high-level (OE18) overexpression lines compared with moderate-level (OE14) LbDREB6 plans under drought stress for 5 days. (A) ABP19a. (B) TIP2-1. (C) PIP2-8. (D) GolS. (E) LEA. (F) Peroxidase 18. Data are presented as means of three biological replicates, and error bars represent SD. Asterisks indicate significant differences, *P< 0.05 and **P< 0.01.
