# Supplementary material for: Overexpression Levels of LbDREB6 Differentially Affect Growth, Drought, and Disease Tolerance in Poplar
Source: Front Plant Sci. 2020 Nov 11;11:528550. doi: 10.3389/fpls.2020.528550 (PMC7693672; doi:10.3389/fpls.2020.528550)
Supplement: Supplementary Table 1 — Primers used in qRT-PCR analysis for genes responsive to drought. [file Table_5.DOC]

Supplementary table S1 Primers used in qRT-PCR analysis for genes responsive to drought.

| Genes name | Accession number | Primer sequences (5’-3’) | Size of PCR fragment (bp) |
| --- | --- | --- | --- |
| *PuActin* | MH644084 | F: AGGGTATGCCCTTCCACAT  R: TCAAGGGCAACATATGCAAG | 115 |
| *Aquaporin TIP2-1* | Potri.003G050900 | F: CCTCTTATTGGTGGAGGGCT  R: TTAGAACTCATAGGACGAAG | 84 |
| *Aquaporin TIP1-1* | Potri.006G121700 | F: GTGAGCTGGACCTGGACCAA  R: TTAGTAGTCAGCGGTCGGCA | 115 |
| *Peroxidase 15* | Potri.005G112200 | F: TACGCAGAGGATGAGGATGC  R: CATAGAAGTAACTTAGGATT | 184 |
| *Peroxidase 3* | Potri.007G122100 | F: ATGAGGGGGTTTCGTTATTT  R: TATGATAGCTGCTGCCAGTG | 180 |
| *Peroxidase 18* | Potri.018G015500 | F: GCCGCAAATGTGAGACC  R: GGTCTCACATTTGCGGC | 204 |
| *ABA8′OH* | Potri.004G235400 | F: GGAGAAGAAACAAGGGAGTG  R: CACTCCCTTGTTTCTTCTCC | 143 |
| *NPF 5.8* | Potri.017G152800 | F: ATGGCCAGTGGGCAGAAACC  R: GGATGTGAATCCACACCAAT | 186 |
| *SHINE-2* | Potri.006G253800 | F: ATGAGGGTCGAGCTTGGAAA  R: CTCAGGTTCAATCTCAACTC | 108 |
| *GST-1* | Potri.011G140400 | F: ATGGCTGATGAGGTGACACT  R: AGTTGGGAGCTGCTTGGATA | 180 |
| *GST-2* | Potri.011G140600 | F: ATGGCTGATGAGGTGACACT  R: ATTGTGGATGAGAGTTGGGA | 180 |
| *WRKY 49* | Potri.016G099900 | F: CCCACTCCAAGGAATGAAG  R: CTTCATTCCTTGGAGTGGG | 203 |
| *DREB2C* | Potri.009G128500 | F: ATGGCAGGAAACGCAGGAGT  R: TACGATAGTGTCCATTGCAA | 234 |
| *Peroxidase 6* | Potri.014G157500 | F: AGGAGGCACCCTTATCCTTC  R: GTCGATACTATCCAAGGGTC | 152 |
| *ATHB-13* | Potri.010G093400 | F: AGCTGCAAGAATTTGACACA  R: ATTACTGCTAGAACCCTCTG | 114 |
| NFYA | Potri.009G030800 | F: ATGTCTTGCTGTGGAGGAAA  R: CACGTACATCCTAACTGGAG | 153 |
| WRKY 40 | Potri.003G182200 | F: CCATTACTCTTGATCTCACA  R: TCACCACTTCTCGCTGTGAT | 200 |
| *WRKY 46* | Potri.002G168700 | F: TGACCTATCGAGGGAGGCAT  R: GGAGCCCAAGTCCTCGTTTT | 197 |
| *ABP19a* | Potri.001G169000 | F: TCCTCCCATGCAGCTGATTT  R:CAACGCCAAGGCCAGTAAAC | 124 |
| *PIP2-8* | Potri.005G109300 | F: AGGTTTCGCTGATTCGAGC  R:GCCATCCGTTTAGGATCGGT | 224 |
| *GolS* | Potri.010G150400 | F: TCGAGCCCACGTTTAACTCC  R:ACACTGGCTCAATCTCACGG | 213 |
| *LEA* | Potri.018G052500 | F: CCCTTCACGGTCTCTGCATT  R:ACGTCAAGATCGTTCGACCC |  |

*Supplementary table S2 Primers used in qRT-PCR analysis for disease resistance genes.*

| Genes name | Accession number | Primer sequences (5’-3’) | Size of PCR fragment (bp) |
| --- | --- | --- | --- |
| *PuActin* | Potri.019G010400.1 | F: AGGGTATGCCCTTCCACAT  R: TCAAGGGCAACATATGCAAG | 115 |
| *PP2C-1* | Potri.006G105000.1 | F:TCCCGTCCAACTCACTGTTG  R:GAGTTGAAATGAGGCCGT | 205 |
| *PP2C-2* | Potri.001G092100.1 | F:GGTGGCGAAGGAAGTGGAG  R:TTTATTGACAACGCTTCC | 203 |
| *NB-LRR1* | Potri.017G152400.1 | F: AAGTGGGCTTGGAAATGAC  R:GAATGCTTCCTTGGATTTG | 115 |
| *NB-LRR2* | Potri.005G041300.1 | F:CGAAAGTCTTGCTCAACTCG  R:TTCCCTACCATCAGCACTTTC | 188 |
| *NB-LRR3* | Potri.T092600.1 | F:GGCATGGCAACCTTAATGCC  R:GGATGGAATAGCAGTAGACAC | 197 |
| *RPS2* | Potri.005G042000.1 | F:GAAGGTCAGCAGCCACTTCT  R:CCAATTTAGGGAACCTTGG | 205 |
| *RPM1-1* | Potri.T093200.1 | F:AGTTCGGCGTCTATCAATCC  R:GGTAGGAAGGTGGAAAGGG | 184 |
| *RPM1-2* | Potri.005G007700.1 | F:TCGTTCTTCCCTGACACTC  R:TTCTCTTAGCTAAAATGCCC | 177 |
| *PYL4* | Potri.006G104100.1 | F:CGCCCACGCTGTAGGCCCC  R:GGAGAGTACCCACGTCACCGT | 168 |
| *ABP19A-1* | Potri.003G065300.1 | F:AGCTTTTGTACAGCAATTTC  R:TAAACAGTGTTGGCCG | 170 |
| *ABP19A-2* | Potri.001G169000.1 | F:CAGCAATTTCCCGGTG  R:TAACATCACCCTTCTTAAG | 177 |
| *WRKY49* | Potri.016G099900.1 | F:GGCTGGTGATGGTTAT  R:TGAAATGGTCCTCTT | 196 |
| *WRKY9* | Potri.005G141400.1 | F:CCATGCGCGCTTTAGAAG  R:CCTCTCAATCTCCCCGT | 253 |
| *NR* | Potri.009G101600.1 | F:GGGAAAGATGGTTGGGCAG  R:TACATTCCACTTCCTTGGCCT | 199 |
| *GA20ox1* | Potri.004G146000.1 | F:GGGGAATGGATTCGAGTT  R:TTGTCAGCTCCTCCAGGGGC | 163 |

Supplementary table S3 Statistical analysis of sequencing.

| **Samples** | **Clean reads** | **Clean bases** | **GC content** | **%≥Q30** |
| --- | --- | --- | --- | --- |
| **A1** | 24,724,618 | 7,333,341,242 | 44.48% | 96.58% |
| **A2** | 17,708,048 | 5,241,601,974 | 44.20% | 96.61% |
| **A3** | 18,719,483 | 5,540,725,368 | 44.63% | 96.33% |
| **B1** | 19,819,054 | 5,870,785,184 | 44.31% | 96.59% |
| **B2** | 20,483,146 | 6,062,187,306 | 44.49% | 96.67% |
| **B3** | 20,527,149 | 6,099,446,068 | 45.57% | 95.02% |
| **C1** | 18,613,427 | 5,504,766,388 | 44.08% | 96.71% |
| **C2** | 18,206,408 | 5,365,976,912 | 44.42% | 96.79% |
| **C3** | 20,801,834 | 6,176,570,496 | 45.32% | 96.56% |

Note: A1-A3 wild type plants, B1-B3 *LbDREB6-MO* transgenic plant line, C1-C3 *LbDREB6-HO* transgenic plant line.
